# Supplementary material for: On the role of initial velocities in pair dispersion in a microfluidic chaotic flow
Source: Nat Commun. 2017 Sep 7;8:468. doi: 10.1038/s41467-017-00389-8 (PMC5589773; doi:10.1038/s41467-017-00389-8)
Supplement: Supplementary file 1 — Supplementary information [file 41467_2017_389_MOESM1_ESM.pdf]

Description of Supplementary Files

File name: Supplementary Information
Description: Supplementary figures, supplementary notes and supplementary references.

File name: Peer Review File

Supplementary Information

Supplementary Figures

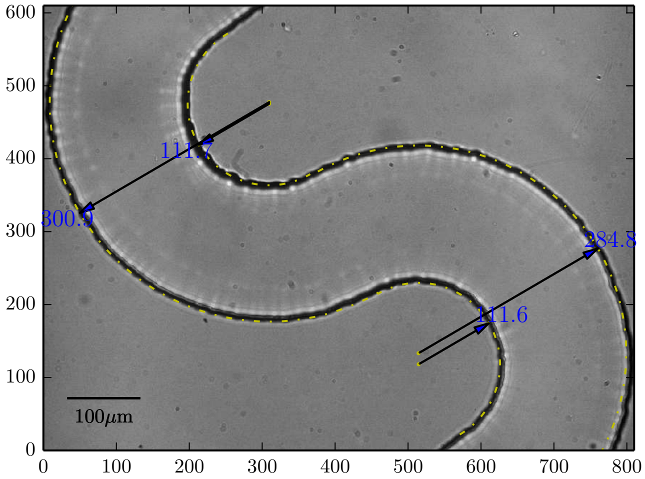

Supplementary Figure 1: **Microfluidic tube geometry.** A bright field image of the observation window; the tube geometry is based on a planar concatenation of co-centric pairs of half circles; the observation window encloses two out of 33 in total; the arrows, and the yellow broken line indicate the tube walls and their dimensions, specified in microns; the tube has a rectangular cross-section of 140 µm depth (perpendicular to the image plane).

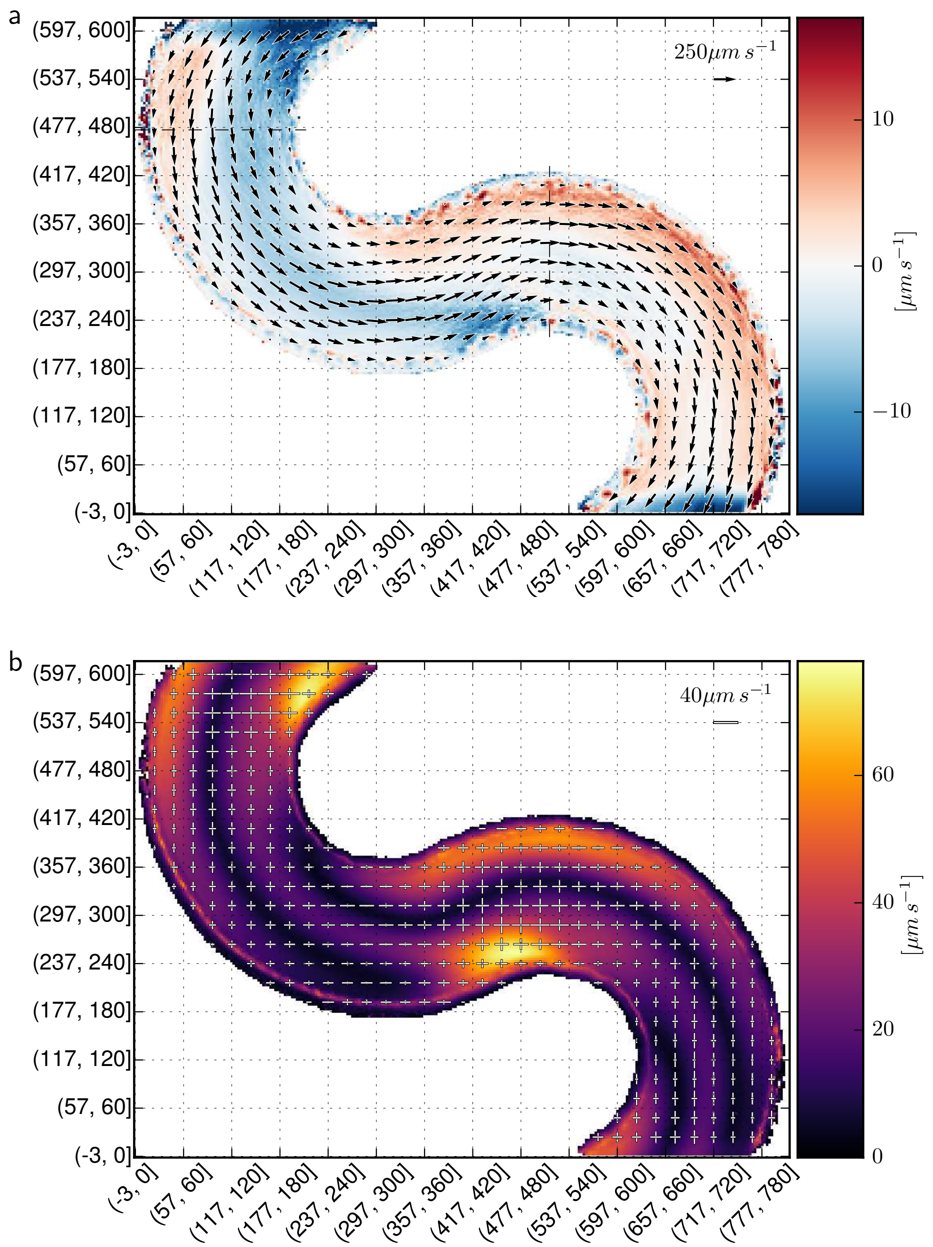

Supplementary Figure 2: **Spatial Eulerian statistics of the velocity field: mean value and standard-deviation over time.** The transformation from the Lagrangian frame of reference to the Eulerian picture was achieved by subdividing the inner volume of the channel to a grid of $3\,\mu m \times 3\,\mu m \times 3\,\mu m$ units; data points along tracer trajectories were assigned to an Eulerian coordinate whenever they passed in the corresponding grid unit. The mean and standard-deviation of the velocity components for each unit sample were calculated. Here we present a selection of cross-sections along the channel, where the out-of-plane velocity component statistic is encoded in the colourmap; the standard-deviation of the in-plane components are represented by line lengths. (**a**) & (**b**) show the velocity field at mid-channel depth, mean and standard-deviation correspondingly; for the sake of visualisation, the in-plane components are shown every eighth grid unit. The presence of an out of plane mean flow indicates an appreciable deviation from laminar Poiseuille-like flow; the cross-sections in what follows, corresponding to the two black dashed lines in (**a**), further reveal the non-Poiseuille nature of the mean flow.

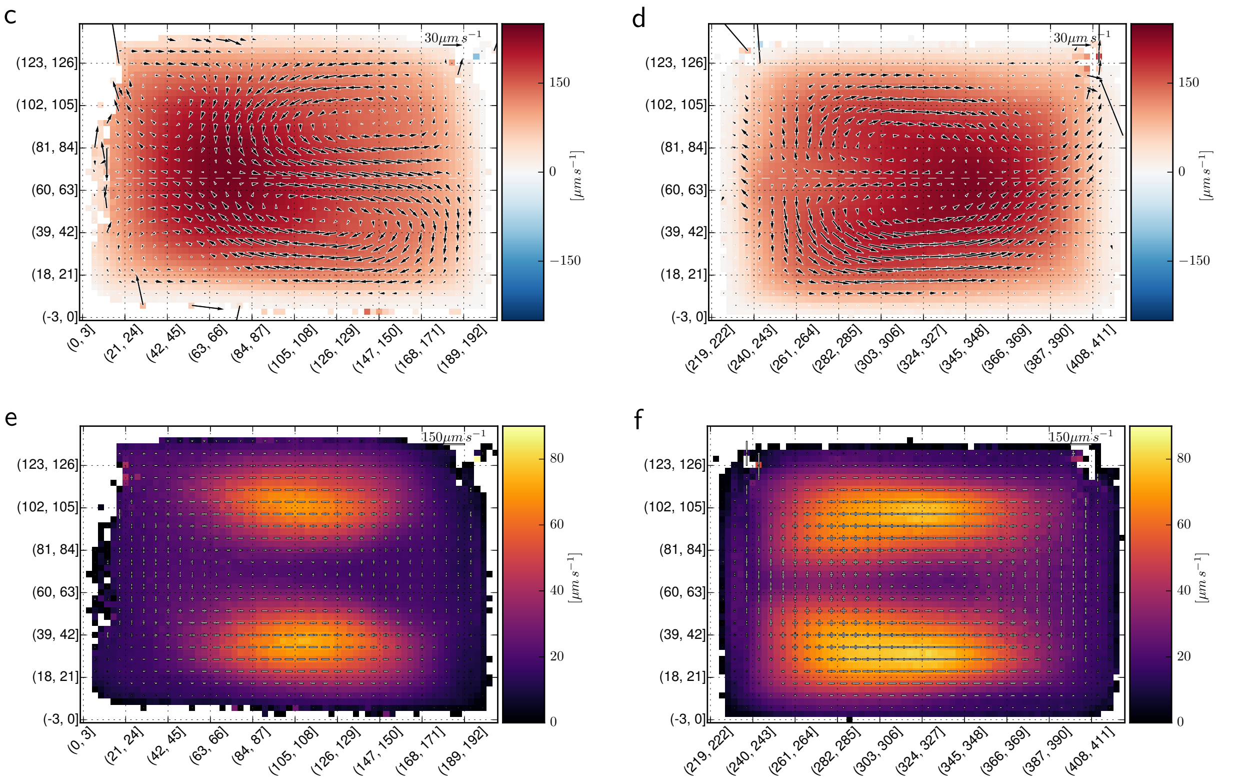

Supplementary Figure 2: **Spatial Eulerian statistics of the velocity field (*continued*).** (**c**) & (**e**) present the velocity field on a radial-vertical cross-section of the curvilinear channel, along the upstream dashed line in Supplementary Figure 2a, mean and standard-deviation correspondingly; (**d**) & (**f**) show the corresponding data for the downstream cross-section; the white dashed lines in c & d denotes the mid-channel depth, across which Supplementary Figure 2a & b is taken, as well as the stream-wise component profiles to follow. Both radial-vertical cross-section fields reveal two main helical flows (circulating arrows; plotted every second grid unit to ease visualisation) centred near the inner wall, and a stream-wise component (colourmap) peak located mid-way between them and shifted radially towards the external wall; here inner and external refer to centres of the half circles which form the tube, as shown in Supplementary Figure 1. The fluctuation intensities, as measured by the standard-deviation fields, show higher values above and below the mid-depth line, reaching values comparable to the mean velocity itself in extended regions. The non-zero radial and vertical components themselves are in strong contrast to what is expected had it been a laminar Poiseuille-like flow, where these should vanish altogether.

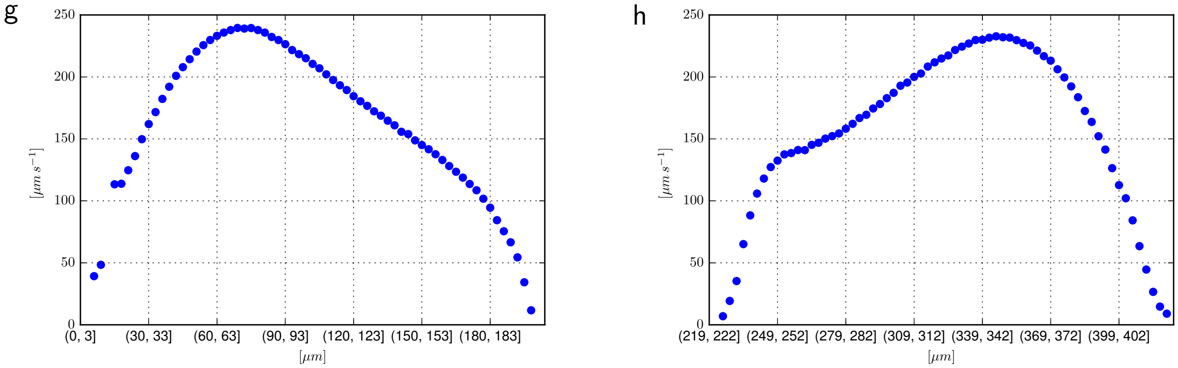

Supplementary Figure 2: **Spatial Eulerian statistics of the velocity field (*continued*).** (**g**) & (**h**) show the stream-wise velocity component profile as function of the radial direction, along the mid-depth cuts, denoted by dashed white lines in Supplementary Figure 2c & d, correspondingly. As expected from developed elastic turbulence under similar geometry, these show high resemblence to the profile presented in [1, Fig. 10]; as can be seen, time-averaged longitudinal velocity exhibit a non-Poiseuille-like characteristic of an approximately linear profile over a significant fraction of the channel width, attributed to the efficient diffusion of momentum due to the mixing properties of elastic turbulence. These stand in sharp contrast with those found under the same conditions, only when polymers are absent, resulting in a laminar Poiseuille-like profile; see [1, Fig. 2], where the laminar profile persisted even at values of the Reynolds number which were three orders of magnitude larger compared with the one in this study.

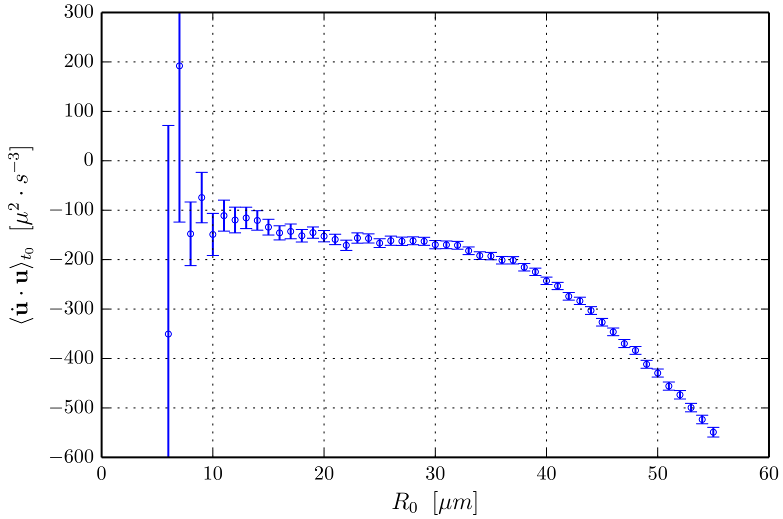

Supplementary Figure 3: **The coefficient of the cubic term in Equation 2, $\langle \dot{\mathbf{u}} \cdot \mathbf{u} \rangle_{R_0,t_0}$.** The averages are taken at t_0, when the pairs separation distance is closest to R_0, as function of R_0, for $\langle \dot{\mathbf{u}} \cdot \mathbf{u} \rangle_{R_0,t_0}$. It is also an estimator for the time derivative of $\langle \frac{1}{2} u^2 \rangle_{R_0,t_0}$. The error bars indicate the margin of error based on the 95% confidence.

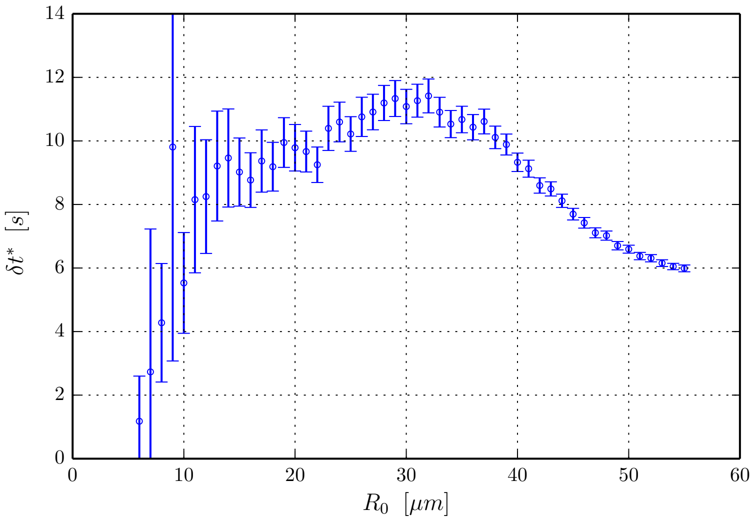

Supplementary Figure 4: **Transition time from the ballistic regime.**
$\delta t^*_{R_0} = \left| \langle u^2 \rangle_{R_0,t_0} \Big/ \langle \dot{\mathbf{u}} \cdot \mathbf{u} \rangle_{R_0,t_0} \right|$ is plotted as function of the initial separation distance R_0. Deviations of the relative dispersions $\langle \| \mathbf{R} - \mathbf{R_0} \|^2 \rangle_{R_0}$ from the δt^2 scaling are generically expected to be significant for $\delta t \gtrsim 0.1\, \delta t^*$.

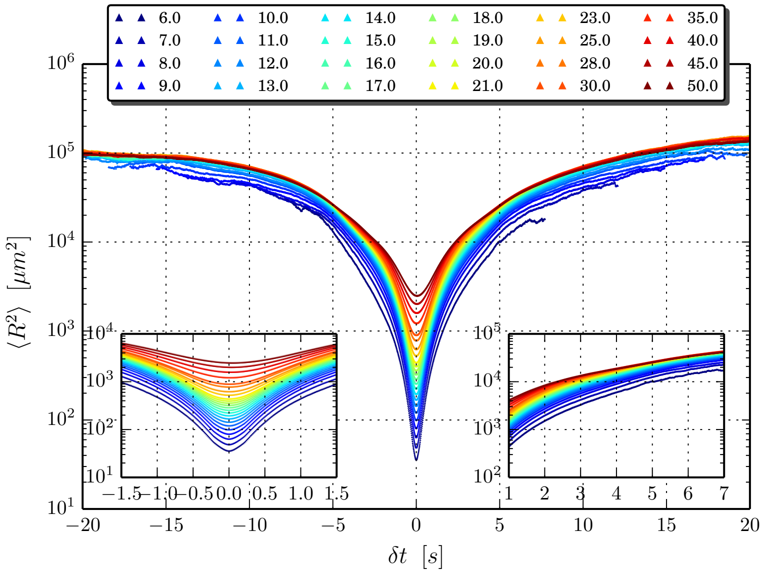

Supplementary Figure 5: **Pair dispersion.** The plot shows the average squared pair separation distance, $\langle R^2 \rangle_{R_0}$ for various R_0 between 6 and 50 µm; the insets show a zoom-in on the initial and intermediate time intervals. This is the same data plotted in the main text Figure 2, only here not normalised by R_0.

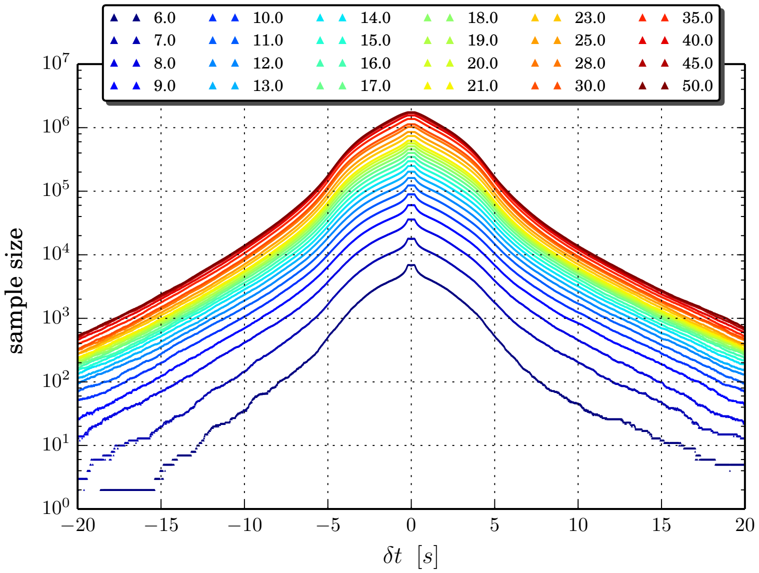

Supplementary Figure 6: **Sample sizes.** The number of pairs in the datasets presented in this work plotted against δt for the various initial separation distances R_0. These sample sizes result from several filters on the observed pairs: each single trajectory is required to span more than 140 µm; every $R(t)$ trajectory is let to contribute to an R_0 bin at most once, at the point where it is nearest to the centre of the bin, this moment is denoted t_0 for the pair in this bin; the pair trajectory is required to contain at least 15 measurement points before and after t_0. Averages of sample sizes smaller than 100 were excluded.

Supplementary Note 1: Exponential pair dispersion – theoretical prediction

The study of particle dispersion in flows is at the basis of the understanding of transport processes [2, 3, 4]. In the early 1950s, G. K. Batchelor predicted that the mean length of material lines in turbulent flows would grow exponentially in the course of time, in the long time limit; this stems from the notion that the line elements it consists of can be considered short enough such that the distance between the ends of an element remain within the dissipative scale throughout the motion [5, 6]. In some recent works the terms 'material line' and 'material line element' are used interchangeably to indicate the separation vector between two passive particles in the fluid [7, 8, 4]. And indeed Batchelor's prediction has been later reformulated for tracer particles in the form of exponential pair separation; see [4, §2] for example. To illustrate how this comes about, let us consider a pair of passive tracers separated by the vector $\mathbf{R}$, and whose relative velocity is $\mathbf{u}$. The evolution of the squared separation distance R^2 follows

$$\frac{1}{2}\frac{d}{dt}R^2 = \mathbf{u}\cdot\mathbf{R} = u_l R \ , \tag{1}$$

where u_l is the separation velocity, defined by the above relation. The analysis then proceeds by assuming a linear flow approximation — the pair separation is considered to be small enough such that the velocity of one tracer is linearly related to that of the other. Using this approximation Supplementary Equation 1 reduces to

$$\frac{1}{2}\frac{d}{dt}R^2 = \tilde{\xi}R^2 \ , \tag{2}$$

where $\tilde{\xi}$ no longer depends on R. For the case of chaotic flows, $\tilde{\xi}$ can be modelled as a random variable, and analyses often focus on the expectation value of such equations [6, 2, 4]. Additionally assuming the correlation time of $\tilde{\xi}$ to be very short compared to the observation time [2, 4], a generalisation of the central limit theorem – the multiplicative ergodic theorem of Oseledec [2, 9] – is applied, resulting in the exponential pair dispersion

$$\langle R(t)^2\rangle = R^2(t_0)\,\exp\left\{2\xi\,(t-t_0)\right\} \ . \tag{3}$$

This is a relation for the time evolution of the second moment of pair separation distances. In this form one can identify 2ξ with the generalised Lyapunov exponent of the second order, which is generically not trivially related to the ordinary (maximal) Lyapunov exponent; see [10, §3.2.1], [9, §5.3], [2] and others.

Much of the theoretical and numerical literature discussing pair dispersion in the dissipative sub-range is devoted to the evaluation of ξ in terms of the typical time-scale of the flow τ, that is $\xi = \gamma/\tau$; see [5, Eq. 2.9]. The value of γ is still under debate as can be learnt from [6, §24.5] as well as [4] and references therein.

Supplementary Note 2: Smoothness of elastic turbulence – experimental evidence

As we recall in the main text, the common theoretical framework employed to analyse elastic turbulence relies on the assumption that the flow is smooth in space [11, 12, 13]. In this work we have demonstrated that the linear velocity field approximation does not hold at scales beyond a rather small fraction of the apparatus — less than 10% of the tube linear width in our case; see

Figure 3. This may seem at odds with previous experimental reports [14, 15, 1]. Yet, we find no contradiction.

Based on the velocity power spectrum presented in [15, Fig. 23], Burghelea *et al.* reported a power-law decaying faster than -3, in support of the notion of a spatially smooth flow. However the lower cut-off for that scaling corresponds to scales smaller than a third of the smallest spatial scale of the apparatus, limiting the extent of the implications.

The results presented in [1, Fig. 28–29 & 33–34] are as restrictive as ours. There the scaling of structure functions changes before 10% of the apparatus size is reached.

One consequence of our findings is that the Lyapunov exponents picture [14, 16, 12] is not the appropriate one to describe the dynamics of wall-bounded elastic turbulence at scales much larger than few percent of the vessel size. Indeed, Burghelea *et al.* [14] performed a Finite Time Lyapunov Exponent analysis on numerically integrated particles (similarly to Jullien [17]), only one has to recognise that their results do not demonstrate that in their experiment pairs diverged exponentially in time.

This raises questions regarding the mechanism for polymer stretching once their end-to-end distance goes beyond, as in our case. In fact, Smith *et al.* [18] have shown that a steady shear flow is sufficient to stretch polymers to about 40% of their full length.

Supplementary Note 3: Exponential pair dispersion – experimental evidence

Literature review

At the time of writing, the exponential pair dispersion, briefly presented in Supplementary Note 1, is regarded as the leading paradigm for chaotic flows which are spatially smooth, as manifested by the analysis of recent experimental results and the discussions which follow.

Jullien [17] studied an instance of the Batchelor regime flow in two-dimensional turbulence, where the velocity field was inferred experimentally followed by numerical integration of tracers simulated on a computer; the initial pair separation values were set to distances smaller than the measurements grid. An exponential separation, referred to in that context as Lin's law [19], was reported during an intermediate time interval of between one to twice the value of the estimated flow typical time scale τ, after which a power-law scaling has been observed.

Salazar and Collins [4] estimated γ $(= \xi\tau)$ from measurements of $\langle\tilde{\xi}\rangle$ in three-dimensional turbulence reported by Guala *et al.* [8]; this estimate should be taken with a grain of salt not only because it is unclear whether these measurements were indeed restricted to the dissipative scales but also as, although related, the quantities $\langle\tilde{\xi}\rangle$ and ξ are not the same [6].

Even more recently, Ni and Xia [20] reported measurements in three-dimensional turbulent thermal convection and inferred γ from exponential fits to the mean squared pair separation distance; as presented in [20, Fig. 1], the fits are taken at time intervals of up to one Kolmogorov time-scale, a time too short with respect to the underlying assumptions, and thereafter the data grows faster than the evaluated exponentials. Additionally, as we mention in the main text, for an asymptotic exponential pair dispersion to be demonstrated, the various curves should all have the same exponential rate and differ only by the initial separation; such a result has not been empirically demonstrated.

We therefore find it safe to conclude that the literature on the subject is lacking conclusive experimental evidence.

Discussion in the light of this study

Studying pair separation in the dissipative sub-range over long times in intense turbulence poses a technological challenge. The high velocities, typical of high Reynolds number flows, restrict the length of the obtained trajectories as exemplified by the above mentioned reports [17, 7, 20] and other recent works [21]. This is one of the reasons for which the experimental literature on pair dispersion in smooth chaotic flows is lagging behind the theoretical one.

Recall that the exponential growth in Supplementary Equation 3 relies on two underlying assumptions [5, 2, 4]: (i) the velocity field admits a linear approximation in space throughout the observation time; and (ii) the observation time is much longer than the correlation time of the velocity gradients. These requirements are quite stringent and are clearly not fulfilled by our experiment. To the best of our knowledge these assumptions have not been met experimentally for tracer particles so far, and yet the exponential growth prediction seems to be the leading paradigm in interpreting experimental results [17, 4, 20]. Our estimations for the experimental system presented here indicate these may be possibly relevant for $R_0 \lesssim 0.1\,\mu\text{m}$ and $\delta t \gtrsim 10\,\text{s}$. Examining the experimental parameters reported in recent works [3, 22, 21] we find that the trade-off between short correlation times and a large enough dissipation scale renders them difficult to reach in intense inertial turbulence.

Following the above discussion one may reach the conclusion that the Batchelor prediction [5] is irrelevant. By all means, this is not the case! Going back to Batchelor's own words:

> *"In that paper no consideration was given to the particular case of two particles which are so close together – or of clouds whose linear dimensions are so small – that the value of the spatial derivative of the velocity is the same at the (simultaneous) positions of the particles, for the reason that such a condition is unlikely to be realized with practical methods of marking and observing particular fluid particles.*
>
> ...
>
> *Difficulties of observation of marked particles which are very close together do not worry us in the present connexion, since we are concerned here with the changes in the total length of a material line, which is made up of the changes in a large number of infinitesimal line elements, unlike the changes in the shortest distance between two fluid particles."*
>
> — see G. K. Batchelor, *Proc. R. Soc. A* 1952 [5]

We find that Batchelor himself appreciated how challenging would his assumptions be when it comes to finite size particles. At the same time, it is important to note that his prediction addresses the evolution of the total length of a material line, where each sub-segment could always be taken infinitesimally small, unlike the case of the shortest distance between pairs of tracers starting with a finite initial separation.

Supplementary References

[1] Jun, Y. & Steinberg, V. Elastic turbulence in a curvilinear channel flow. *Phys. Rev. E* **84** (2011).

[2] Falkovich, G., Gawędzki, K. & Vergassola, M. Particles and fields in fluid turbulence. *Rev. Mod. Phys.* **73**, 913–975 (2001).

[3] Bourgoin, M., Ouellette, N. T., Xu, H., Berg, J. & Bodenschatz, E. The role of pair dispersion in turbulent flow. *Science* **311**, 835–838 (2006).

[4] Salazar, J. P. & Collins, L. R. Two-particle dispersion in isotropic turbulent flows. *Annu. Rev. Fluid Mech.* **41**, 405–432 (2009).

[5] Batchelor, G. K. The effect of homogeneous turbulence on material lines and surfaces. *Proc. R. Soc. A***213**, 349–366 (1952).

[6] Monin, A. S. & Yaglom, A. M. *Statistical Fluid Mechanics, Volume II: Mechanics of Turbulence (Dover Books on Physics)*, vol. 2 (Dover Publications, 2007).

[7] Lüthi, B., Tsinober, A. & Kinzelbach, W. Lagrangian measurement of vorticity dynamics in turbulent flow. *J. Fluid Mech.* **528**, 87–118 (2005).

[8] Guala, M., Lüthi, B., Liberzon, A., Tsinober, A. & Kinzelbach, W. On the evolution of material lines and vorticity in homogeneous turbulence. *J. Fluid Mech.* **533** (2005).

[9] Cencini, M., Cecconi, F. & Vulpiani, A. *Chaos: From Simple Models to Complex Systems.* Series on advances in statistical mechanics (World Scientific, 2010).

[10] Paladin, G. & Vulpiani, A. Anomalous scaling laws in multifractal objects. *Phys. Rep.* **156**, 147–225 (1987).

[11] Fouxon, A. & Lebedev, V. Spectra of turbulence in dilute polymer solutions. *Phys. Fluids***15**, 2060 (2003).

[12] Berti, S., Bistagnino, A., Boffetta, G., Celani, A. & Musacchio, S. Two-dimensional elastic turbulence. *Phys. Rev. E* **77** (2008).

[13] Steinberg, V. Elastic stresses in random flow of a dilute polymer solution and the turbulent drag reduction problem. *C R Phys***10**, 728–739 (2009).

[14] Burghelea, T., Segre, E. & Steinberg, V. Statistics of particle pair separations in the elastic turbulent flow of a dilute polymer solution. *Europhys. Lett.***68**, 529–535 (2004).

[15] Burghelea, T., Segre, E. & Steinberg, V. Elastic turbulence in von karman swirling flow between two disks. *Phys. Fluids***19**, 053104 (2007).

[16] Gerashchenko, S., Chevallard, C. & Steinberg, V. Single polymer dynamics: coil-stretch transition in a random flow. *Europhys. Lett.***71**, 221–227 (2005).

[17] Jullien, M.-C. Dispersion of passive tracers in the direct enstrophy cascade: Experimental observations. *Phys. Fluids***15**, 2228 (2003).

[18] Smith, D. E. Single-polymer dynamics in steady shear flow. *Science* **283**, 1724–1727 (1999).

[19] Lin, J.-T. Relative dispersion in the enstrophy-cascading inertial range of homogeneous two-dimensional turbulence. *J. Atmos. Sci.* **29**, 394–396 (1972).

[20] Ni, R. & Xia, K.-Q. Experimental investigation of pair dispersion with small initial separation in convective turbulent flows. *Phys. Rev. E* **87** (2013).

[21] Bewley, G. P., Saw, E.-W. & Bodenschatz, E. Observation of the sling effect. *New J. Phys.* **15**, 083051 (2013).

[22] Ouellette, N. T., Xu, H., Bourgoin, M. & Bodenschatz, E. An experimental study of turbulent relative dispersion models. *New J. Phys.* **8**, 109 (2006).
